# A Rectangular Notch-Band UWB Antenna with Controllable Notched Bandwidth and Centre Frequency

**DOI:** 10.3390/s20030777

**Published:** 2020-01-31

**Authors:** Anees Abbas, Niamat Hussain, Min-Joo Jeong, Jiwoong Park, Kook Sun Shin, Taejoon Kim, Nam Kim

**Affiliations:** Departement of Computer and Communication Engineering, Chungbuk National University, Cheongju 28644, Korea; anees@chungbuk.ac.kr (A.A.); hussain@osp.chungbuk.ac.kr (N.H.); mjhouse1102@gmail.com (M.-J.J.); jwpark@osp.chungbuk.ac.kr (J.P.); kammul@hanmail.net (K.S.S.); ktjcc@cbnu.ac.kr (T.K.)

**Keywords:** UWB antenna, rectangular notch, electromagnetic bandgap (EBG), notch-band antenna

## Abstract

This paper presents the design and realization of a compact ultra-wideband (UWB) antenna with a rectangular notch wireless area network (WLAN) band that has controllable notched bandwidth and center frequency. The UWB characteristics of the antenna are achieved by truncating the lower ends of the rectangular microstrip patch, and the notch characteristics are obtained by using electromagnetic bandgap (EBG) structures. EBGs consist of two rectangular metallic conductors loaded on the back of the radiator, which is connected to the patch by shorting pins. A rectangular notch at the WLAN band with high selectivity is realized by tuning the individual resonant frequencies of the EBGs and merging them. Furthermore, the results show that the bandwidth and frequency of the rectangular notch band could be controlled according to the on-demand rejection band applications. In the demonstration, the rectangular notch band was shifted to X-band satellite communication by tuning the EBG parameters. The simulated and measured results show that the proposed antenna has an operational bandwidth from 3.1–12.5 GHz for |S_11_| < -10 with a rectangular notch band from 5–6 GHz, thus rejecting WLAN band signals. The antenna also has additional advantages: the overall size of the compact antenna is 16 × 25 × 1.52 mm^3^ and it has stable gain and radiation patterns.

## 1. Introduction

During the last two decades, ultra-wideband (UWB) technology has attracted the attention of both academics and industries. UWB is a technology that uses a very low energy level for short-range, high-information spread over a large bandwidth, usually more than 500 MHz. Due to the short duration of UWB pulses, it is easier to engineer high data rates with low latency. This encourages the usage of UWB in sensor networks, wireless positioning systems, biomedical imaging, and high data short-range communications [1].

Antennas have a major effect on the performance of UWB communication systems, therefore, the design of an antenna must meet its typical requirements, such as impedance matching, radiation stability, compact size, and low cost, which is quite challenging to achieve. The patch antennas are good candidates for UWB applications because of their lightweight, planar geometry, and ease of integration with other electronic components [2,3].

The main challenge in UWB communication is to avoid existing licensed and unlicensed wireless communication bands within the UWB spectrum, such as local area networks (WLAN) (IEEE802.11a, HIPERLAN/2,) operating in the 5.15–5.825 GHz band, WiMax (3.3–3.6 GHz), and X-band satellite communication (7.25–8.395 GHz). Filters are usually used to reject unwanted bands to improve communication quality. However, the use of filters increases the cost and volume of the system as well as insertion losses [4,5]. Therefore, much research has been focused on the design of UWB antennas that have band rejection features to avoid potential interference from existing bands. Previous studies have developed many notched band UWB antennas using different approaches to avoid interference problems. These band rejection techniques include etching slots on patch [6,7,8], split-ring resonators [9,10,11], electromagnetic bandgap (EBG) structures [12,13,14], and inserting a resonant cell in a microstrip line [15,16]. Some researchers achieved notched band capabilities by coupling parasitic elements to the radiator [17,18].

**Figure 1:**
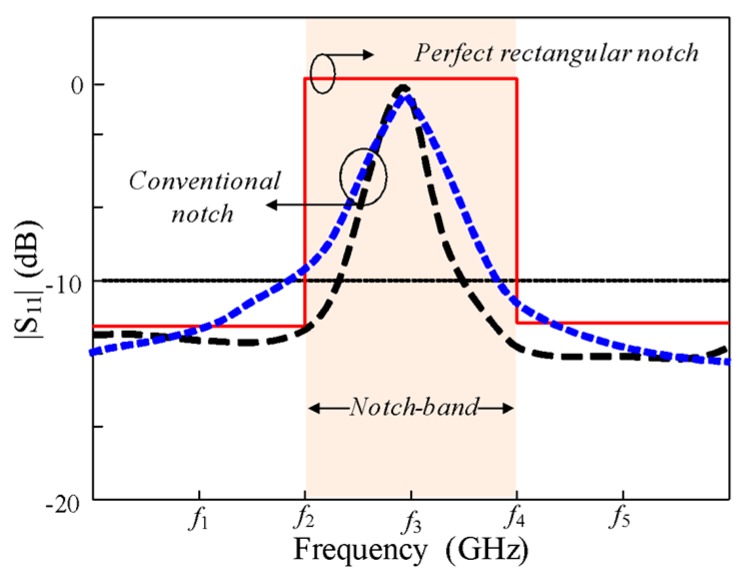
Conventional notch and the rectangular notch**.**

These techniques facilitate the design of notch-band antennas. However, References [6,7,8,9,10,11,12,13,14,15,16,17,18] designs had the common disadvantage of a conventional notch with poor selectivity. The conventional notch is only capable of rejecting interference in the central frequency of a targeted band. To notch the unwanted bands in practical applications, a rectangular notch with high selectivity is required. Figure 1 shows a typical conventional notch and rectangular notch bands. 

In recent years, few rectangular notched antennas have been reported, because of their potential to reject the unwanted bands [19,20,21,22,23,24]. In [19], using a large antenna, two conventional notch bands were attained at 5.2 and 5.8 GHz to reject the wireless local area network WLAN (5–6 GHz) without filtering the bandwidth between two bands. In [20], a rectangular notch-band antenna is presented at the WLAN band; however, the size of the antenna is too large to be used in modern compact electronic devices. In [21], several slots and split-ring resonators were coupled to a Coplanar Waveguide (CPW) line to achieve a wideband notch**;** however, because of its complicated geometry, the antenna does not offer frequency shifting of the notch band. In [22], to achieve a sharp-selectivity notch band, multiple quarter-wavelength slits were etched on the ground, and half-wavelength stubs were coupled to a microstrip; however, the geometry of the antenna was too complicated and required two-port excitations for its operation. In [23], folded strips and two pairs of inductively coupled resonators were employed for band-rejection with the advantage of controllable bandwidth. However, the geometry of the antenna was complex, and the overall size of the antenna is large. In [24], many slots and split-ring resonators were used to realize a notch with sharp selectivity with controllable bandwidth; however, because of the complex geometry, the notch-band frequency cannot be shifted. Hence, the design of a compact rectangular notch UWB antenna with controllable notch bandwidth and frequency is needed.

This paper presents the design and realization of a compact UWB microstrip patch antenna with a rectangular notch band having controllable notch bandwidth and frequency. Two EBGs are used to realize the rectangular notch at the WLAN band. Moreover, we demonstrate that notch bandwidth and frequency could be controlled according to the application requirements by tuning the parameters of the EBG.

## 2. Antenna Design

### 2.1. Antenna Structure

**Figure 2:**
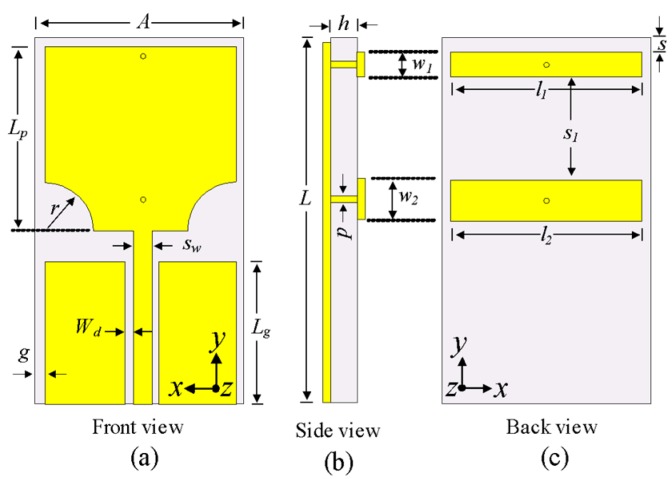
Geometry of proposed antenna: (**a**) front view (**b**) side view, and (**c**) back view.

The geometry of the proposed antenna is shown in Figure 2. The antenna consists of a modified rectangular patch and two EBG structures. The lower corners of the patch are truncated by a semicircle with radius *R *to achieve UWB characteristics. The EBG structures consist of two rectangular conductors that are connected to the radiator using two shorting pins with radius *p*. The EBGs are usually made up of small metal patches on dielectric substrates with shorting vias and have the potential to create a stopband to block electromagnetic waves of a certain frequency [25]. The motive of using EBG structure is the ease of design, and simplification of the antenna. The antenna is printed on Taconic TLY-5A substrate (ε_r_ = 2.17 and tanδ = 0.0009) with length *L *and width *A*. The antenna is fed with a coplanar waveguide with a characteristic impedance of 50 Ω. The optimized dimensions of the antenna are as follows: *A *= 16 mm,* L* = 25 mm,* h* = 1.52 mm, *Lg *= 7 mm, *Lp* = 12 mm,* r* = 0.3 mm, *Sw* = 3.2 mm, *Wd* = 0.6 mm, *l_1_*= 16 mm,* l_2_* = 16 mm, *w_1_* = 0.9 mm, *w_2_*= 2 mm, *s *= 1.1 mm, *g *= 1 mm, and *p *= 0.2 mm.

### 2.2. Design of the UWB Antenna

**Figure 3:**
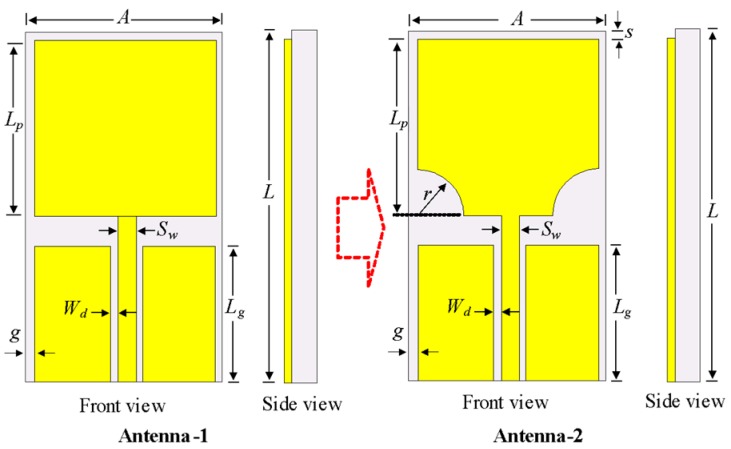
The design evolution of the ultra-wideband patch antenna.

The design of the UWB antenna evolved from the simulation of a conventional rectangular patch antenna (Antenna-1) that is fed with a coplanar waveguide in CST Microwave Studio. Because conventional monopoles have a narrow bandwidth, the lower ends of Antenna-1 were truncated in a semicircle with radius *R* to achieve UWB characteristics. Figure 3 shows the evolution of the UWB patch antenna design, which is designated as Antenna-2.

The optimized parameters of the Antenna-1: *A* = 16 mm, *L* = 24 mm, *h* = 1.52 mm, *g* = 1 mm, *Lg* = 7 mm, *Lp* =12 mm, *Sw* = 3.2 mm, and *Wd* = 0.6 mm. The optimized parameters of the Antenna-2: *A* = 16 mm, *L* = 24 mm, *h* = 1.52 mm, *g* = 1 mm, *Lg *= 7 mm, *Lp* =12 mm, *Sw* = 3.2 mm, *Wd* =0.6 mm, and *r* = 0.3 mm.

**Figure 4:**
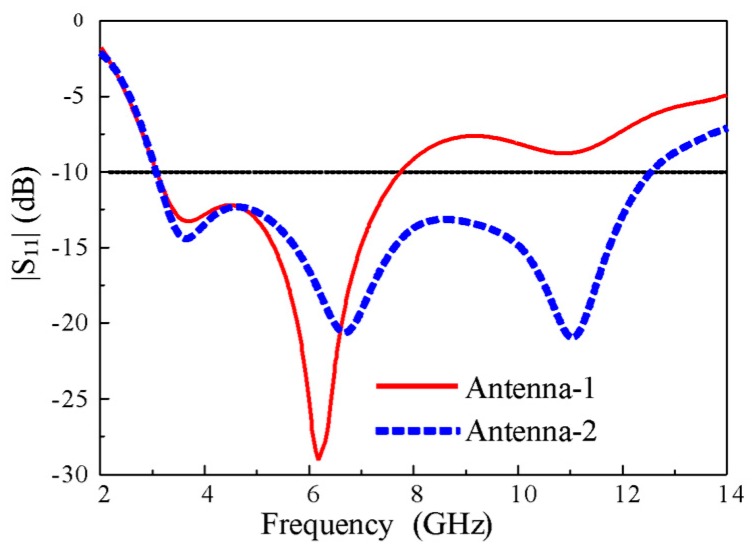
|S_11_| characteristics of Antenna-1 and Antenna-2.

The impedance characteristics of both antennas in terms of |S_11_| are shown in Figure 4. The Antenna-1 showed an impedance bandwidth ranging from 3.1–7.8 GHz for |S_11_| < -10 dB, which is dramatically increased from 3.1 to 12.5 GHz in Antenna-2. It is worth noting that the impedance bandwidth of the antenna covered the entire bandwidth required for UWB technology, which is proposed by the Federal Communication Commission (FCC). 

### 2.3. Design of The UWB Antenna with A Conventional Notch

**Figure 5:**
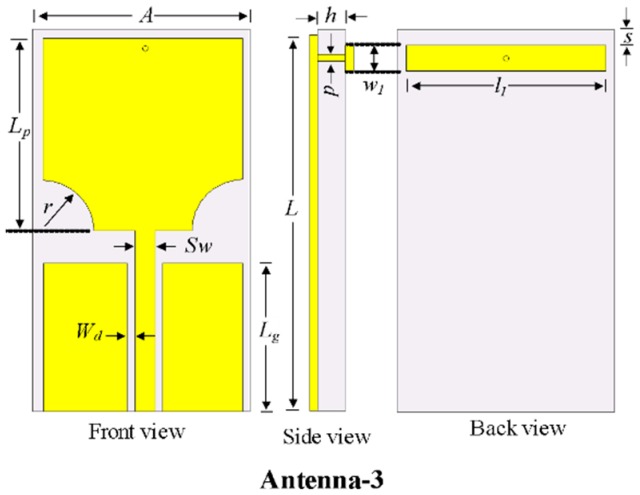
Design procedure to achieve conventional notched UWB patch antenna.

**Figure 6:**
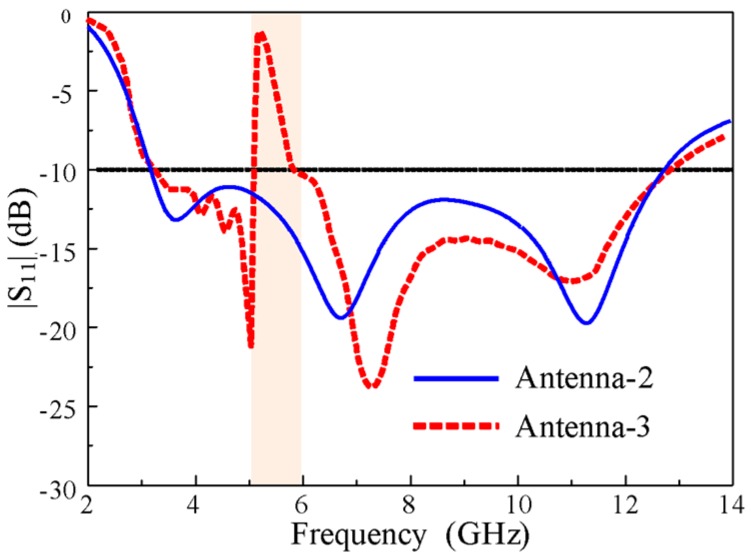
|S_11_| characteristics of Antenna-2 and Antenna-3**.**

The notch characteristics are realized in UWB antennas to reject signals from unwanted bands using EBG structures. To achieve the notch characteristics in the UWB antenna (i.e., Antenna-2), a rectangular EBG was introduced on the back of the substrate, which is connected to the patch by a shorting pin, as shown in Figure 5 and designated as Antenna-3. Figure 6 shows a comparison of the |S_11_| characteristics of Antenna-2 and Antenna-3. The antenna with the one optimized EBG achieved a conventional notch at the WLAN band (5.15–5.9 GHz) without degrading the overall bandwidth of the UWB antenna. Thus, a notch was successfully realized.

The optimized parameters of the Antenna-3: *A* = 16 mm,* L* = 24 mm,* h* = 1.52 mm, *g* = 1 mm, *Lg* = 7 mm, *Lp* = 12 mm, *Sw* = 3.2 mm, *Wd* = 0.6 mm,* r* = 0.3 mm, *l_1_* = 16 mm, *w_1_* = 0.9 mm, *s* = 1.1 mm, and *p* = 0.2 mm.

### 2.4. Design of the UWB Antenna with the Rectangular Notch

**Figure 7:**
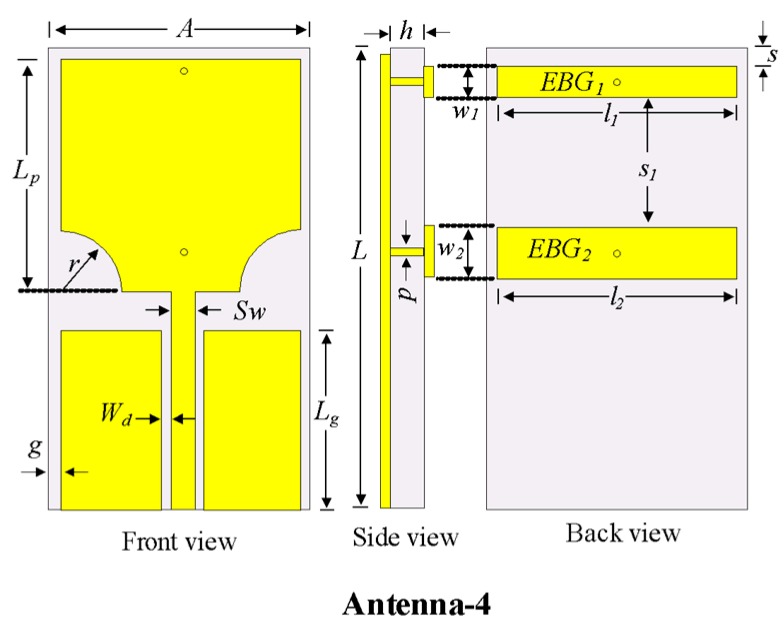
Characteristics of Antenna-2 and antenna design procedure to achieve conventional notched UWB patch antenna.

**Figure 8:**
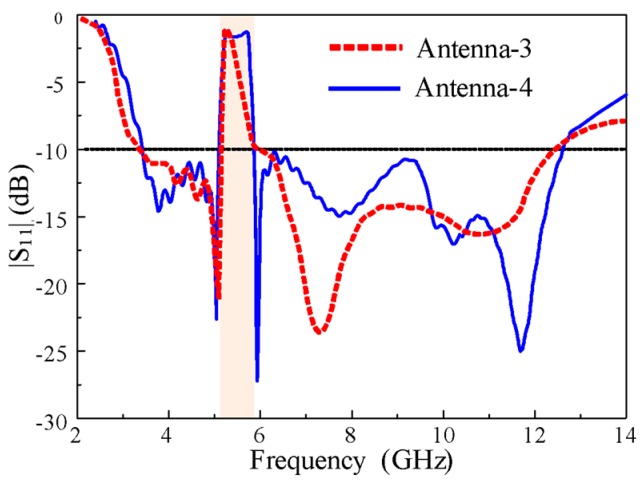
|S_11_| characteristics of Antenna-3 and Antenna-4.

The conventional notch using one EBG described in the previous section had high selectivity at lower frequencies but low selectivity at higher frequencies. Therefore, another EBG was added to Antenna-3 and optimized to obtain high selectivity in the entire rejection band by merging the individual notch bands of EBG1 and EBG2. Figure 7 shows the geometry of the rectangular notch-band antenna with EBG1 and EBG2 (i.e., Antenna-4). The |S_11_| characteristics of Antenna-3 and Antenna-4 are shown in Figure 8. The conventional notch of Antenna-3 (with one EBG) was changed to a rectangular notch band with high selectivity in Antenna-4 (with two EBGs). The important parameters that affected the notch-band characteristics are discussed later in this paper. 

**Figure 9:**
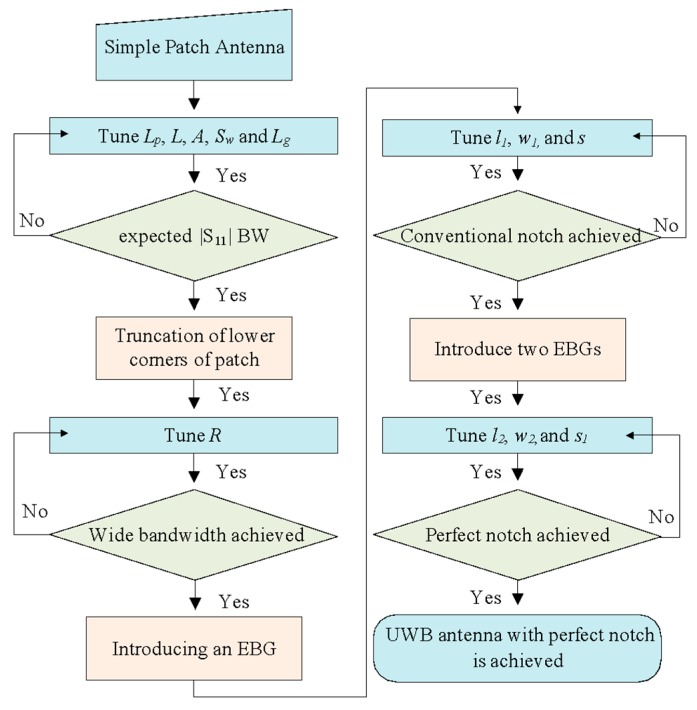
Design procedure of the proposed antenna with rectangular notch-band characteristics.

The design summary of the rectangular notch UWB antenna is illustrated in the detailed flowchart shown in Figure 9. First, a simple rectangular patch that is fed with a microstrip line with the partial ground plan is designed. The antenna is then optimized by varying the parameters of the antenna. The lower ends of the patch are then truncated in the semicircle *R *and tuned to attain UWB characteristics. A rectangular EBG is introduced, and its parameters (*l_1_, w_1_,* and* s*) are optimized to realize a conventional notched band. Finally, another EBG is designed and its parameters (*l_2,_ w_2_*, and *s_1_*) are tuned to achieve a rectangular notch band in the UWB antenna.

### 2.5. Controllable Notch Bandwidth and Frequency

**Figure 10:**
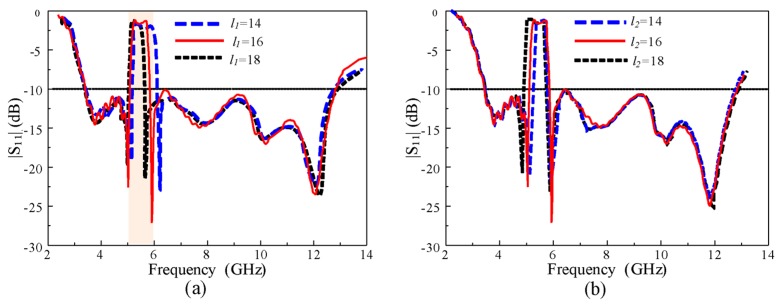
|S_11_| of the antenna for different lengths of: (**a**) EBG1* l_1_*, and (**b**) EBG2 *l_2_.*

The bandwidth of the rejection band is an important characteristic of the design of UWB antennas. The notch bandwidth and frequency should be adjusted according to the requirements of the wireless communication system. In the proposed antenna, the length and width of both EBG structures are key parameters that affect the notch bandwidth of the antenna. To make it clear, we are studying the parametric study. It is to be noted that during the parametric study, only one parameter is changed, and all other parameters are unchanged. Figure 10a shows the |S_11_| of the antenna according to different lengths (*l_1_*) of EBG1. The upper frequency of the notch band shifted linearly toward the left side, increasing the *l_1_*without affecting the passband of the antenna. Because EBG1 controls the upper frequency of the notch, the lower frequency showed almost no change in the *l_1_.* However, the length *(l_2_*) of EBG2 affects the lower frequency of the notch band, as shown in Figure 10b. The lower frequency of the notch shifted toward the left side, increasing (*l_2_*) while showing a negligible change in the upper frequency of the notch band. 

**Figure 11:**
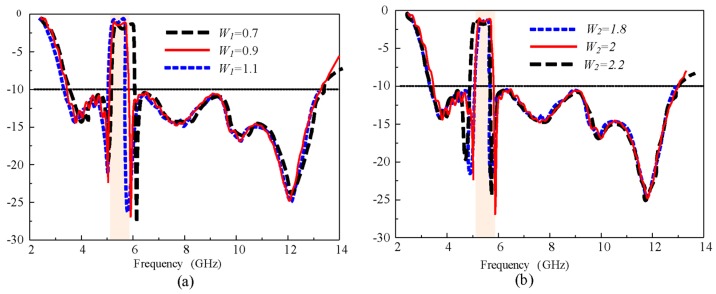
|S_11_| of the antenna for different widths of (**a**) EBG1 *w_1_* and (**b**) EBG2 *w_2_.*

**Figure 12:**
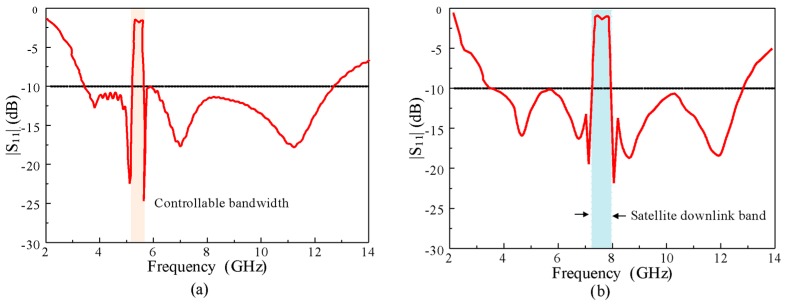
Demonstration of controllable notch band of the proposed antenna: (**a**) bandwidth and (**b**) frequency.

Figure 11 shows the effect of changing the EBG widths on the |S_11_| of the antenna. The variations in the width of EBG1 (*w_1_*) and EBG2 (*w_2_*) show a response similar to the change in the length of the EBG. The upper frequency of the notch band moved to the left side, increasing the values of *w_1_*, and *w_2_* changed the lower frequency of the notch band in the same way as *w_1_*. These observations indicate that by tuning the dimensions of the EBGs, we can control the bandwidth and notch frequency of the proposed antenna according to the demands of the application. In the demonstration, the bandwidth of the notch band was reduced from 5–6 to 5.2–5.8 GHz, and the notch band was shifted to the X-band satellite downlink (7–8 GHz), as shown in Figure 12. For the reduction (5.2–5.8 GHz) in the bandwidth of the notch band *l_1_*was increased by 2 mm, while* l_2_* was decreased by 2 mm from the optimized values. Moreover, to shift the notch from WLAN to the X-band, the width, length, and spacing between the EBGs were reoptimized. The optimized parameters of the X-band notch antenna are as follows: *A* = 16 mm, *L *= 24 mm, *h* = 1.52 mm, *g* = 1 mm, *Lg *= 7 mm, *Lp* =12 mm, S*w* = 3.2 mm, *Wd* = 0.6 mm, *r *= 0.3 mm,* l_1_* = 8.5 mm, *w_1_*= 2 mm, *s* = 3.5 mm, and *p* = 0.2 mm, *l_2_* = 8.5 mm, *w_2_* = 2, and *s_1_* = 11.3 mm.

## 3. Antenna Measurements

**Figure 13:**
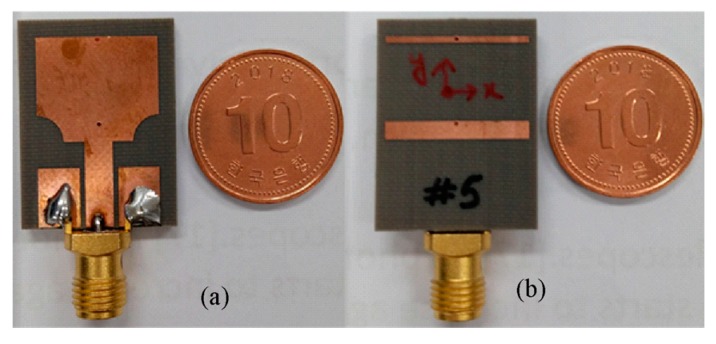
Photo of fabricated UWB antenna with rectangular notched band: (**a**) front side (**b**) back side.

**Figure 14:**
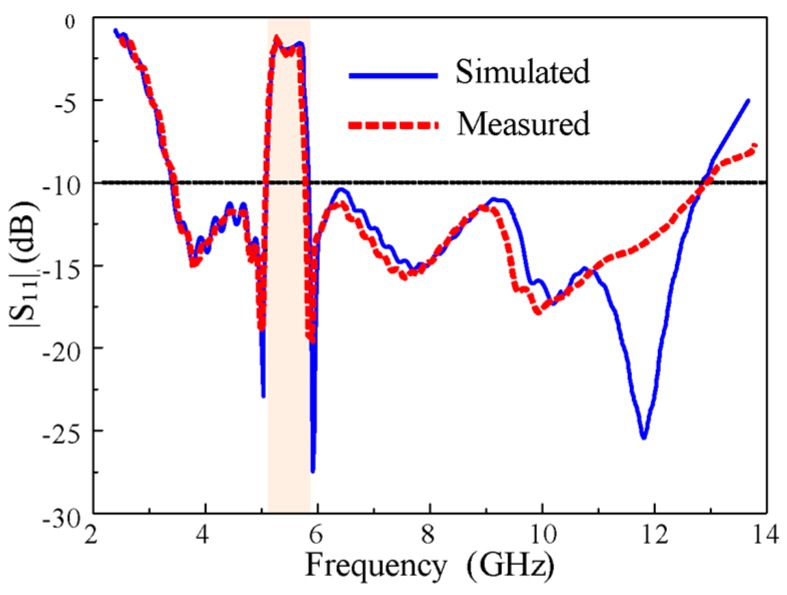
|S_11_| characteristics of the proposed antenna.

The modeled antenna was fabricated on Taconic TLY 5A (1.52 mm) for experimental verification, as shown in Figure 13. The impedance and radiation characteristics of the designed antenna were measured by an Agilent vector network. Figure 14 shows the simulated and measured impedance characteristics. The measured and simulated results are well-matched. The antenna showed good impedance matching characteristics throughout the entire UWB spectrum (3.1–12.5 GHz) with the exception of the WLAN band (5.1–5.9 GHz), which was rejected to avoid interference. There is a difference between simulated and measurement |S_11_| results, especially at high frequencies. This discrepancy is most likely due to cable losses and fabrication tolerances.

**Figure 15:**
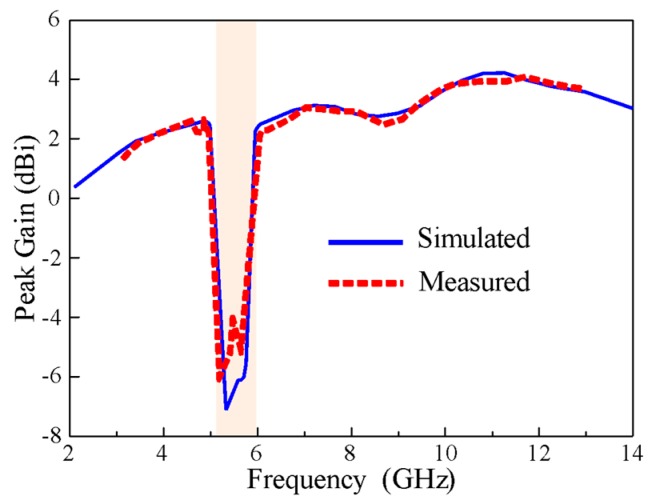
The measured and simulated gain of the proposed antenna.

The measured and simulated peak gains of the antenna are plotted as a function of frequency, as shown in Figure 15. In the measurement of peak gain, a well-calibrated standard horn antenna was used as the source antenna while the fabricated prototype was measured as the receiving antenna. Transmit and receiver amplifiers were used to provide stable power reception. The antenna under test was rotated to measure the peak gain at different orientations. A stable peak gain curve was obtained in the frequency range of interest with a maximum value of 4.5 dBi, but it sharply decreased to about -6 dBi at the notch band. This demonstrates the effectiveness of the antenna’s rectangular notching characteristics. 

**Figure 16:**
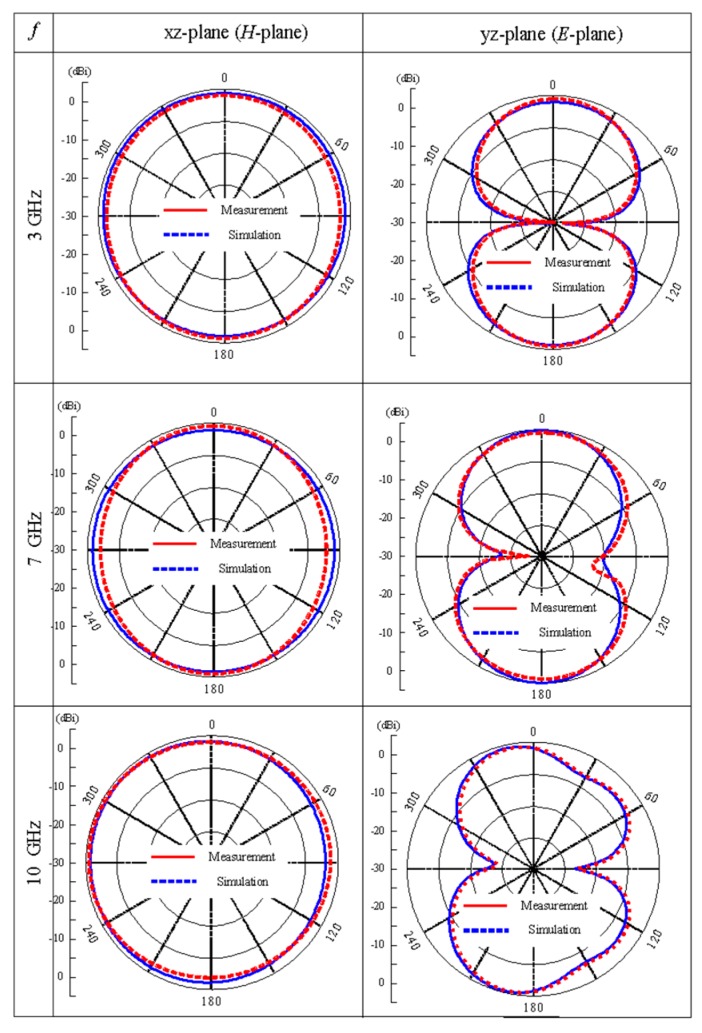
Simulated measure radiation pattern in E-and H-plane radiation pattern at 3, 7, and 10 GHz.

Figure 16 shows the simulated and measured radiation patterns in* H*-plane (xz-plane) and *E*-plane (yz-plane) at 3, 7, and 10 GHz. The radiation pattern at *H*-plane is nearly symmetrical and omnidirectional for 3, 7, and 10 GHz, however, in *E*-plane the radiation patterns are bidirectional with dumbbell shape. The radiation pattern at *E*-plane in 10 GHz shows little deterioration because of the increased radiating area at higher frequencies. 

Finally, the antenna is compared with the previously reported rectangular notched band antennas [19,20,21,22,23,24] in terms of overall size, controllability of the notch-band bandwidth and frequency, and design complexity (Table 1). Almost all the antennas are large and have complex geometries. The antenna presented in [19, 21] are very large and lack controllable frequency and bandwidth. Although the antennas reported in [20, 22] offer the advantages of controllable bandwidth and frequency, the former is very large, and the latter have the disadvantage of design complexity, which is due to the presence of many stubs and slots; moreover, they require two-port excitations in its operation. The antenna designs proposed in [23, 24] offer controllable bandwidth without frequency shifting of the notch band, which is due to their complex geometry. In summary, the proposed antenna outperformed the existing rectangular notch UWB antennas based on its simple design and small size, as well as the controllable bandwidth and frequency of the notch band.

## 4. Conclusion

A rectangular notched band UWB antenna with controllable bandwidth and frequency with an overall compact size of 16 × 25 × 1.52 mm^3^ is presented. The antenna is fabricated on the TLY 5A (ε_r_=2.17) substrate. The proposed antenna covers the entire UWB spectrum from 3.1 to 12.5 GHz. The UWB characteristic of the antenna is achieved by truncating the lower ends of the patch. The conventional notch is obtained by using a single EBG structure, and a rectangular notch at the WLAN band is realized by using two EBG structures with optimized parameters, which are laid on the back of the substrate and connected to the patch by shorting pins. The results showed that the notch band and frequency can be tuned by changing the EBG parameters. The measurement results of the fabricated antennas are well-matched to the simulation results. 

## Figures and Tables

**Table 1:** Performance comparison of the proposed antenna with existing rectangular notched antenna in literature.

Ref. antennas	Antenna size (mm^3^)	Controllable notch-bandwidth	Controllable notch frequency	Design complexity
[19]	44 × 44.4 × 0.1	No	No	simple
[20]	48 × 50 × 1	Yes	Yes	simple
[21]	50 × 50 × 1.575	No	No	complex
[22]	28 ×18 × 0.8	Yes	Yes	very complex
[23]	38 × 42 × 0.5	Yes	No	very complex
[24]	8.5 × 22 × 0.8	Yes	No	complex
This work	16 × 25 × 1.52	Yes	Yes	simple
